# Effects of sevoflurane on lung epithelial permeability in experimental models of acute respiratory distress syndrome

**DOI:** 10.1186/s12967-023-04253-w

**Published:** 2023-06-18

**Authors:** Ruoyang Zhai, Woodys Lenga Ma Bonda, Charlotte Leclaire, Cécile Saint-Béat, Camille Theilliere, Corinne Belville, Randy Coupet, Raiko Blondonnet, Damien Bouvier, Loic Blanchon, Vincent Sapin, Matthieu Jabaudon

**Affiliations:** 1 iGReD, UFR de Médecine et des Professions Paramédicales, Place Henri Dunant, CNRS, INSERM, Université Clermont Auvergne, 63000 Clermont-Ferrand, France; 2 Department of Perioperative Medicine, CHU Clermont-Ferrand, Clermont-Ferrand, France; 3 Department of Medical Biochemistry and Molecular Genetics, CHU Clermont-Ferrand, Clermont-Ferrand, France

**Keywords:** Acute respiratory distress syndrome, Sevoflurane, Lung epithelial barrier function, Junction proteins, Intracellular pathways, Receptor for advanced glycation end-products

## Abstract

**Background:**

Preclinical studies in acute respiratory distress syndrome (ARDS) have suggested that inhaled sevoflurane may have lung-protective effects and clinical trials are ongoing to assess its impact on major clinical outcomes in patients with ARDS. However, the underlying mechanisms of these potential benefits are largely unknown. This investigation focused on the effects of sevoflurane on lung permeability changes after sterile injury and the possible associated mechanisms.

**Methods:**

To investigate whether sevoflurane could decrease lung alveolar epithelial permeability through the Ras homolog family member A (RhoA)/phospho-Myosin Light Chain 2 (Ser19) (pMLC)/filamentous (F)-actin pathway and whether the receptor for advanced glycation end-products (RAGE) may mediate these effects. Lung permeability was assessed in RAGE^−/−^ and littermate wild-type C57BL/6JRj mice on days 0, 1, 2, and 4 after acid injury, alone or followed by exposure at 1% sevoflurane. Cell permeability of mouse lung epithelial cells was assessed after treatment with cytomix (a mixture of TNFɑ, IL-1β, and IFNγ) and/or RAGE antagonist peptide (RAP), alone or followed by exposure at 1% sevoflurane. Levels of zonula occludens-1, E-cadherin, and pMLC were quantified, along with F-actin immunostaining, in both models. RhoA activity was assessed in vitro.

**Results:**

In mice after acid injury, sevoflurane was associated with better arterial oxygenation, decreased alveolar inflammation and histological damage, and non-significantly attenuated the increase in lung permeability. Preserved protein expression of zonula occludens-1 and less increase of pMLC and actin cytoskeletal rearrangement were observed in injured mice treated with sevoflurane. In vitro, sevoflurane markedly decreased electrical resistance and cytokine release of MLE-12 cells, which was associated with higher protein expression of zonula occludens-1. Improved oxygenation levels and attenuated increase in lung permeability and inflammatory response were observed in RAGE^−/−^ mice compared to wild-type mice, but RAGE deletion did not influence the effects of sevoflurane on permeability indices after injury. However, the beneficial effect of sevoflurane previously observed in wild-type mice on day 1 after injury in terms of higher PaO_2_/FiO_2_ and decreased alveolar levels of cytokines was not found in RAGE^−/−^ mice. In vitro, RAP alleviated some of the beneficial effects of sevoflurane on electrical resistance and cytoskeletal rearrangement, which was associated with decreased cytomix-induced RhoA activity.

**Conclusions:**

Sevoflurane decreased injury and restored epithelial barrier function in two in vivo and in vitro models of sterile lung injury, which was associated with increased expression of junction proteins and decreased actin cytoskeletal rearrangement. In vitro findings suggest that sevoflurane may decrease lung epithelial permeability through the RhoA/pMLC/F-actin pathway.

**Graphical Abstract:**

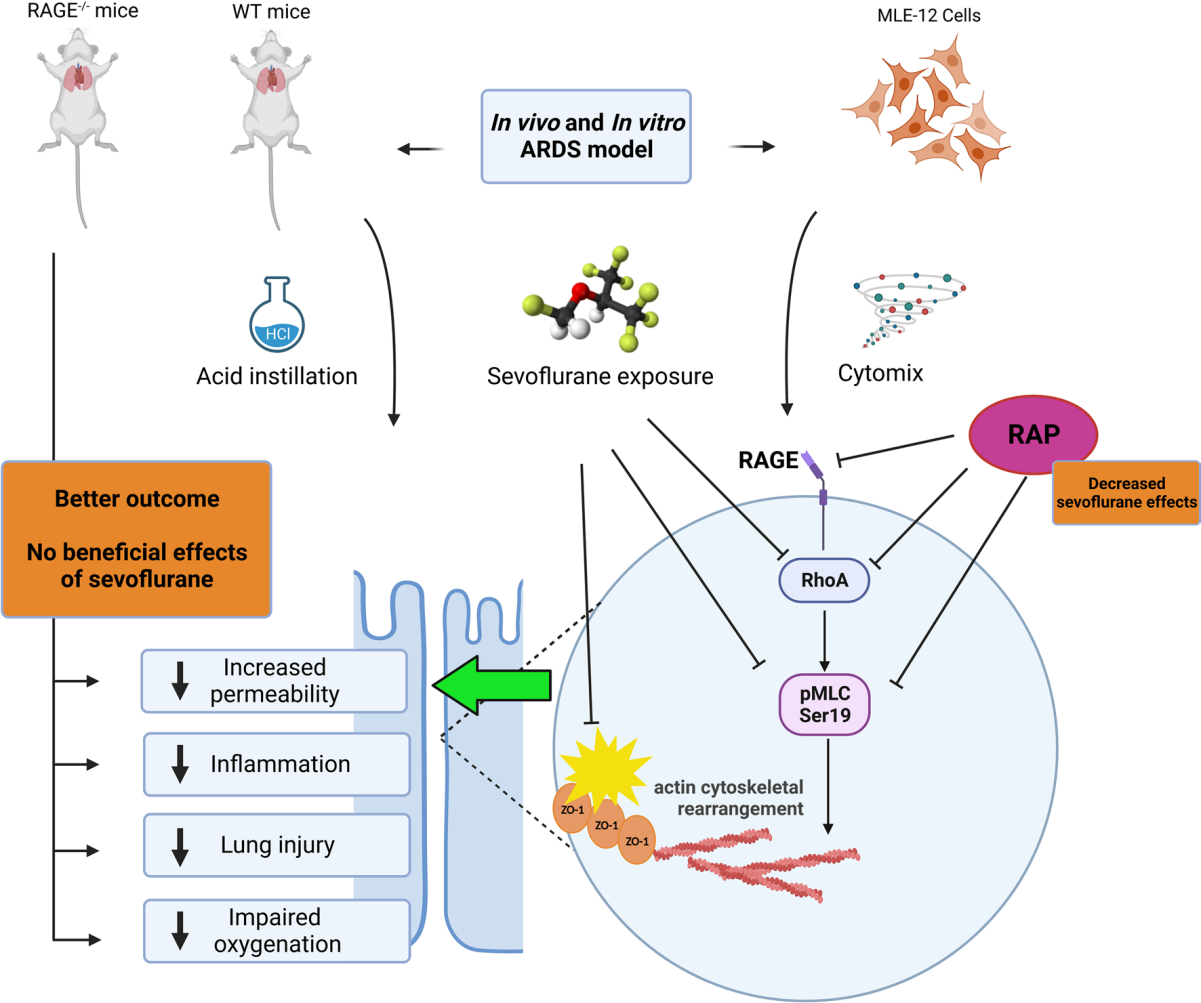

**Supplementary Information:**

The online version contains supplementary material available at 10.1186/s12967-023-04253-w.

## Introduction

Acute respiratory distress syndrome (ARDS) is a clinical syndrome characterized by diffuse alveolar injury, lung edema, and hypoxemic respiratory failure from septic or sterile causes, which frequently occurs in critically ill patients and is associated with a high mortality rate (mortality of 30–50%), greater healthcare utilization, and reduced quality of life or long-term physical and cognitive outcomes among survivors [1–4]. The recent COVID-19 pandemic further highlighted the high morbidity and mortality of ARDS and the high case numbers challenged most healthcare organizations worldwide [5, 6]. Currently, the available treatments for ARDS are largely supportive and based on lung-protective mechanical ventilation, with extracorporeal membrane oxygenation as a rescue option in most severe forms. To date, no pharmacological approach has been successfully translated into clinical practice. Among other mechanisms leading to the accumulation of protein-rich edema fluid in the alveolar spaces, such as endothelial barrier disruption, immune cell recruitment, or thrombo-inflammatory disorders, the degree of injury to the alveolar epithelium is an important determinant of ARDS severity in patients [3]. Epithelial injury includes the dissociation of intercellular junctions with increased paracellular permeability, a process involving the dysregulation of tight junction proteins (e.g., zonula occludens (ZO) proteins) or adherens junction proteins (e.g., E–cadherin) and actin cytoskeletal rearrangement [3, 7–9].

Inhaled halogenated anesthetics, such as isoflurane or sevoflurane, are primarily used for general anesthesia but have gained recent attention for their use in sedation in the intensive care unit [10–12]. Sevoflurane was found to improve gas exchange, reduce alveolar edema, and attenuate pulmonary and systemic inflammation in multiple preclinical models of ARDS [10, 13–17], and one pilot randomized controlled trial in patients with ARDS found that sevoflurane, compared to intravenous midazolam, improved arterial oxygenation and decreased alveolar and plasma levels of some inflammatory cytokines and of soluble receptor for advanced glycation end-products (sRAGE), a marker of lung epithelial injury [18]. Non-randomized evidence suggests potential benefits in patients with ARDS due to COVID-19 [19], and a large multicenter randomized clinical trial is ongoing to assess the impact of inhaled sedation with sevoflurane on clinical outcomes in patients with ARDS [20]. However, the precise mechanisms accounting for the lung-protective properties of sevoflurane remain largely unknown. In a “double hit” mouse model of nebulized lipopolysaccharide (LPS) and ventilator-induced lung injury, isoflurane restored epithelial tight junction integrity via increased ZO-1 protein levels [21], and sevoflurane prevented LPS-induced barrier dysfunction in lung microvascular endothelial cells [22].

To test the hypothesis that sevoflurane could decrease lung epithelial permeability, we used both an in vivo model of acid-induced lung injury in mice and an in vitro model of sterile injury in mouse lung epithelial cells to investigate whether sevoflurane could decrease lung alveolar epithelial permeability through the Ras homolog family member A (RhoA)/phospho-Myosin Light Chain 2 (Ser19) (pMLC)/filamentous (F)-actin pathway. As preclinical studies also reported various potential effects of sevoflurane on the RAGE and RhoA/F-actin pathways in cells of the central nervous system [23, 24] and as the RAGE pathway plays a pivotal role in epithelial injury and repair during ARDS [3, 25–28], we further hypothesized that the effects of sevoflurane on lung epithelial permeability could be, at least partially, mediated by RAGE.

## Materials and methods

### Mouse model of acid-induced lung injury

Animals were maintained and all procedures were performed in the animal facility at University Clermont Auvergne with the approval of the ethics committee of the French *Ministère de l’Education Nationale, de l’Enseignement Supérieur et de la Recherche* (Approval number CE 67-12). The experiments were performed in accordance with relevant regulations, the 3R principles (Replacement, Reduction, and Refinement), and the “Animal Research: Reporting In Vivo Experiments” (ARRIVE) guidelines 2.0 [29].

Female C57BL/6JRj littermate control (Janvier Labs, Saint-Berthevin, France) and RAGE^−/−^ mice (kindly provided by Prof. Ann Marie Schmidt, NYU Langone Health, New York, USA), aged 10–12 weeks and weighing 25–30 g, were anesthetized via an intraperitoneal injection of ketamine (100 mg/kg) and xylazine (10 mg/kg) and given a subcutaneous fluid bolus of 10 μL/g 0.9% isotonic saline as preemptive resuscitation. As previously described [27, 30, 31], 75 μL of a 322 mOsm/L solution (iso-osmolar to mouse plasma) of 0.1 M hydrochloric acid (pH 1.0) was instilled to model ARDS in injured mice. For the next 4 h, mice were kept in a transparent recovery box under humidified supplemental oxygen (inspiratory oxygen fraction (FiO2), reduced gradually from 1.0 to 0.21) and carefully monitored. Their body temperature was maintained using external heat sources, after which they were transferred to individually ventilated cages with air and free access to food and water.

To examine the effects of sevoflurane, lung-injured wild-type and RAGE^−/−^ mice were divided into a Sham group, an HCl group, and an HCl + Sevo group. In the intervention groups, sevoflurane 1% was delivered for 1 h and its ambient concentration was maintained using a gas monitor (AMG-06, Sedana Medical, Danderyd, Sweden). This dose of sevoflurane was considered clinically relevant as an expired fraction of around 1% can provide deep sedation, which is often required in the early management of patients with ARDS [18, 32].

### Physiological measurements in vivo

The criteria for experimental ARDS were evaluated as recommended by the American Thoracic Society [33], at baseline (day 0) in injured and sham animals, and at specified time-points (days 1, 2, and 4) after acid-induced injury [27, 31]. Animals were ventilated for 30 min using volume-controlled ventilation with a tidal volume of 6 µL g^−1^, a positive end-expiratory pressure of 6 cmH_2_O, a respiratory rate of 160 per minute, an inspiration-to-expiration ratio of 1:2, a FiO_2_ of 1.0 (VentElite, Harvard Apparatus, Cambridge, USA). At the end of ventilation, the mice were sacrificed via anesthetic overdose with intraperitoneal pentobarbital (150 µg g^−1^), and arterial blood was sampled for blood gas analysis (Epoc® Blood Analysis System, Siemens Healthineers, Erlangen, Germany), bronchoalveolar lavage (BAL) was performed with 750 μL of saline, and lungs were harvested for molecular biology and histology examination. Acid-injured animals were compared with sham mice, receiving only surgical preparation and 30 min of ventilation. One hour before sacrifice, 10 µg g^−1^ of human serum albumin (HSA) dissolved in 100 μL of saline was retro-orbitally injected for the measurement of the lung permeability index, defined as the ratio of HSA in the BAL fluid to that in the plasma collected at the end of the experiments (human albumin ELISA Kit, R&D Systems, Minneapolis, MN) [31]. In some mice, instead of HSA, a fluorescent tracer IRDye^®^ 800CW (LI-COR Biosciences, Lincoln, USA) was administered retro-orbitally (1 nmol in 100 µL) to visualize and quantify its accumulation in isolated lung samples, and Eppendorf tubes collecting BAL fluid samples (Pearl^®^ Trilogy Small Animal Imaging System, LI-COR Biosciences, Lincoln, USA).

### Cell culture

Virus-transformed murine lung epithelial (MLE-12) cells were obtained from the American Type Culture Collection (CRL-2110™, ATCC, Manassas, USA). The cells were maintained in Gibco Dulbecco's Modified Eagle Medium/Nutrient Mixture F12 (DMEM F12, a 1:1 mixture of DMEM and Ham's F-12) (Thermo Fisher Scientific, Waltham, USA) supplemented with 2% fetal bovine serum (FBS) (Thermo Fisher Scientific, Waltham, USA), 1% penicillin–streptomycin-amphotericin (Eurobio Scientific, Les Ulis, France), 10 nM hydrocortisone and 10 nM β-estradiol (Sigma-Aldrich, St. Louis, USA), and 1X insulin-transferrin-selenium (Thermo Fisher Scientific, Waltham, USA). The cells were incubated at 37 °C in a humidified atmosphere containing 5% CO_2_.

### In vitro treatments

To test the response of MLE-12 cells to an injurious, nonseptic stimulus, the cells were treated with cytomix, a mix of 10 ng/mL each of tumor necrosis factor (TNF)-ɑ, interleukin (IL)-1β, and interferon (IFN)γ (R&D Systems, Minneapolis, MN) in serum-free medium [34]. To test the hypothesis that the RAGE pathway could influence the effects of sevoflurane, cells were treated with 12.5 µg.mL^−1^ of a RAGE Antagonist Peptide (RAP) (Sigma-Aldrich, St. Louis, USA). Treatments were initiated after the cells reached a monolayer with 100% confluency, usually 48 h after seeding to allow formation of intercellular junctions. The cells were exposed to cytomix, administered with medium, for up to 24 h in some experiments. RAP was delivered 30 min before treatment with cytomix.

Exposure to sevoflurane in vitro was delivered through a dedicated vaporizer (Vapor 2000, Dräger, Lübeck, Germany) in a standard and sealed incubator (Thermo Fisher Scientific, Waltham, USA) with specific gas scavenging (Flurabsorb, Sedana-Medical, Danderyd, Sweden). Concentrations of sevoflurane were continuously monitored and maintained at 1% inside the incubator (AMG-06, Sedana Medical, Danderyd, Sweden), for up to 24 h in some experiments.

### Cell viability assay

MLE-12 cells with a seeding density of 10,000 cells were cultured on collagen-coated (50 µg.mL^−1^) 96-well arrays and incubated at 37 °C in a humidified atmosphere containing 5% CO_2_ for 24 h. Then, the culture medium was replaced by the same cell medium conditions as the in vitro treatment conditions. Four replicates were made for each measurement, after 6 h of treatment, WST-8 Solution (Abcam, Cambridge, United Kingdom) was added to each well, OD at 460 nm was measured after incubation for 2 h at 37 °C. The absorbance of the blank wells with the medium only is subtracted from the values for those wells with cells. The viability of cells in the medium group was considered as 100%.

### Electric cell-substrate impedance sensing

MLE-12 cells were cultured to confluence on collagen-coated (50 µg.mL^−1^) 96-well arrays overlying electrodes according to the manufacturer’s protocol (96W10df PET, Applied Biophysics, Troy, USA). Alternating current applied to each electrode was used to calculate the resistance of the cell monolayer over 24 h (ECIS^®^ Z-Theta, Applied Biophysics, Troy, USA), which reflects barrier integrity as resistance decreases when the epithelial monolayer is compromised [35].

### RhoA expression and activity measurements in vitro

RhoA activity was determined using a RhoA-specific G-LISA Activation Assay kit (BK124, Cytoskeleton, Denver, USA) following the per manufacturer’s protocol. The results were normalized to the total RhoA level as measured using the Total RhoA ELISA Biochem Kit (BK150, Cytoskeleton, Denver, USA). Active RhoA was determined in duplicate with the same colorimetric RhoA activation assay in all experimental conditions.

### Histological examination in vivo and immunofluorescence

Formalin-fixed paraffin embedded tissue sections (10 µm) from mice were rehydrated and deparaffinized through a series of xylem ethanol baths. The slices were stained with hematoxylin and eosin (Sigma-Aldrich, St. Louis, USA). Histological features of lung injury were scored by one independent expert, blinded to the treatment groups, using a standardized score as previously described [31, 36].

For immunofluorescence studies, non-specific binding sites were blocked with phosphate-buffered saline (PBS)/1% horse serum buffer for 1 h at room temperature. Sections were then incubated with primary antibodies overnight at 2–8 °C in the incubation buffer (1% bovine serum albumin (BSA), 1% normal donkey serum, 0.3% Triton X-100, and 0.01% sodium azide in PBS). Anti-ZO-1 (61–7300, Invitrogen, Waltham, USA) and anti-E-cadherin (Cell Signaling Technology, Danvers, USA) antibodies were diluted at 1/25 and 1/200, respectively. Slices were washed three times on a rocking station for 15 min with PBS and further incubated with secondary anti-rabbit IgG coupled with AlexaFluor^®^ 647 A-21244 (Invitrogen, Waltham, USA) diluted at 1/500 in the incubation buffer. Control slices without primary antibodies were used as negative controls for the nonspecific binding of secondary antibodies.

MLE-12 cells were seeded in eight-well chamber slides (Nunc™ Lab-Tek™ II Chamber Slide™, Thermo Fisher Scientific, Waltham, USA) at a density of 10^5^ per well in complete medium for 72 h at 37 °C and 5% CO_2_. The cells were then exposed to 10 ng/mL cytomix, in the presence or absence of 12.5 µg/mL RAP, in serum-free medium for 6 h before immunostaining. After treatments, cells were washed with PBS, fixed, and permeabilized in 3.7% paraformaldehyde/0.2% Triton X-100 buffer for 10 min at room temperature. Slides were washed three times in PBS before non-specific sites were blocked with a PBS/BSA 3% solution for 30 min. Then, an anti- ZO-1 polyclonal antibody (61–7300, Invitrogen, Waltham, USA) diluted at 1/25 or an anti-E-cadherin rabbit monoclonal antibody (24E10, Cell Signaling Technology, Danvers, USA) diluted at 1/200 were incubated for 1 h. After three additional washes again, the slides were incubated with an anti-rabbit IgG labeled with AlexaFluor^®^ 647 (A-21244, Invitrogen, Waltham, USA) diluted at 1/1000 for 1 h.

Finally, the cells and tissue sections were washed three times with PBS before nuclei staining with 1 µg.mL^−1^ Hoechst 33,258 diluted at 1/10,000 (Sigma-Aldrich, St. Louis, USA) for 10 min before the slides were washed and covered with an anti-fading mounting medium (CitiFluor MWL4-88, Electron Microscopy Sciences, Hatfield, USA). Samples were observed under a fluorescence microscope (Zeiss Axio Imager M2/Colibri7 coupled Axiocam 506 monochrome camera, power supply 232, and ApoTome.2) at 10X magnification and analyzed with ZEN software v2.1 (Zeiss, Oberkochen, Germany). The same exposure time was chosen to compare fluorescence among all conditions (800 ms for ZO-1 and E-cadherin at 10× magnification).

### mRNA and protein quantification

mRNA was extracted from cultured cells or lung tissues with the Nucleospin Kit (Macherey–Nagel), in accordance with the manufacturer’s instructions. Briefly, cells were scratched and approximately 30 mg of tissues were grinded with 2 mL lysis buffer using the Precellys lysing kit (Bertin Technologies, Montigny-le-Bretonneux, France). For grinding, the Precellys Evolution device (Bertin Technologies, Montigny-le-Bretonneux, France) was used for 1.5 min, with intervals of 15 s between each 15 s of burst at 8,500 rpm. After quantification using the DeNovix DS-11 FX spectrophotometer/fluorometer (DeNovix, Wilmington, USA), retro-transcription was done using 1 µg of mRNA following the high-capacity cDNA reverse transcription kit protocol (Applied Biosystems, Waltham, USA). Real-time polymerase chain reaction (PCR) was performed using the SsoAdvanced SyBR Green Supermix kit (Thermo Fisher Scientific, Waltham, USA) and LightCycler 480 (Roche, Basel, Switzerland). Primers for ZO-1, E-cadherin, RAGE and GAPDH were obtained from the PrimePCR SYBR Green Assays systems (Bio-Rad Laboratories, Hercules, USA). LightCycler 480 was programmed for 40 cycles of two-step cycling for 30 s at 95 °C and 30 s at 60 °C, followed by a melting curve and cooling step. To monitor any changes in mRNA levels, we used the 2^−ΔΔCT^ method after normalization with the housekeeper gene GAPDH.

Proteins from treated cells were obtained by scratching cell monolayers with a RIPA buffer containing a mixture of 1X protease inhibitors, 1 mM sodium orthovanadate (Sigma-Aldrich, St. Louis, USA), and 1X PhosSTOP (Roche, Basel, Switzerland). Tissues were grinded in a Precellys lysing kit (Bertin Technologies, Montigny-le-Bretonneux, France) with 2 mL-tubes containing ceramic beads in RIPA buffer. The tubes were first centrifuged at 14,000×*g* for 10 min to remove cell debris and beads. Then, all the lysates were then sonicated for 3 min and centrifuged at 14,000×*g* at 4 °C for 14 min. Supernatant protein concentrations were measured using the BCA assay (Pierce™ BCA protein assay kit (Thermo Fisher Scientific, Waltham, USA). Next, 25 µg of total protein in β-mercapto-ethanol and Laemmli buffer 1X (Bio-Rad Laboratories, Hercules, USA) containing a reducing agent were separated on an SDS-PAGE 4–15% protein gel (Mini-PROTEAN TGX Stain-Free gels, Bio-Rad Laboratories, Hercules, USA), before being transferred to nitrocellulose membranes using the Trans-Blot Turbo Transfer System (Bio-Rad Laboratories, Hercules, USA). The membranes were then saturated for 1 h at room temperature in the TBST buffer (50 mM Tris HCl pH 7.5, 150 mM NaCl, and 0.1% Tween 20, Abcam, Cambridge, United Kingdom) containing 5% of fat-free milk or BSA. Membranes were incubated overnight at 4 °C with primary antibodies. The ZO-1 polyclonal antibody (61–7300, Invitrogen, Waltham, USA) and the anti-E-cadherin rabbit monoclonal antibody (24E10, Cell Signaling Technology, Danvers, USA) were diluted at 1/25 and 1/200, respectively. Antibodies against MLC and pMLC were obtained from the Myosin Light Chain 2 Antibody Sampler Kit #9776 (Cell Signaling Technology, Danvers, USA). After washing with the TBST buffer, the membranes were incubated for 1 h with horseradish peroxidase (HRP)-conjugated secondary anti-rabbit IgG (BI 2407, Abliance, Compiègne, France) diluted at 1/2500–1/5000. Then, the membranes were processed for chemiluminescence detection using Clarity Max ECL Western blotting substrates (Bio-Rad Laboratories, Hercules, USA). Protein detection was performed using a Bio-Rad Imager, and densitometry analysis of protein bands from the Western blot images was performed using Bio-Rad imaging software (Bio-Rad Laboratories, Hercules, USA). The results were normalized to the band intensity in the control condition.

Cytokines (TNF-ɑ, CXCL-1, and IL-6) in the BAL fluid from mice or supernatants from cell cultures were measured using the Ella Automated Immunoassay System (Pro-teinSimple, Bio-Techne, Minneapolis, USA) following the manufacturer’s instructions. Relative fluorescence units were converted to cytokine concentrations using calibration curves provided by the manufacturer. The final results represent the average of triplicate measurements for each analyte. Detection ranges were: 0.43–1810 pg/mL, 0.6–5770 pg/mL, and 0.31–2930 pg/mL for CXCL-1, IL-6, and TNF-ɑ, respectively.

For mouse sRAGE quantification, the Quantikine^®^ ELISA mouse RAGE immunoassay (MRG00, R&D Systems, Minneapolis, USA) was used as per the manufacturer’s instructions. Samples were diluted at 1/10. Measurements of HSA were performed using ELISA (R&D Systems, Minneapolis, MN).

### Statistical analysis

The data analysis was performed using Prism 9 software (GraphPaD software, La Jolla, USA) and Stata version 17 (StataCorp, College Station, USA). Tests were two-sided, with a bilateral type I error set at 5%. Continuous data were expressed as mean ± standard deviation or median and interquartile range depending on their statistical distribution, after evaluating normality using the Shapiro–Wilk test and homoscedasticity using the Fisher–Snedecor test. Continuous parameters were compared between the experimental groups using an analysis of variance or the Kruskal–Wallis test (when t-test assumptions were not met). Random-effects models were used to analyze the longitudinal evolution of the variables, (i) by considering the between- and within-experiment variability (random effects of the subject: intercept and random slope) and (ii) by assessing fixed effects: group, time, and time–group interaction. The normality of the residuals was checked for all models. A limited number of animals was used for baseline comparisons (*n* = 3–4), and 4–6 animals were used in each group on days 1, 2, and 4 [31, 37]. For the cell experiments, three independent series (*n* = 3–4 per series) were performed in duplicate.

## Results

### In vivo effects of sevoflurane on lung injury and alveolar-capillary permeability

Decreased PaO_2_/FiO_2_, increased BAL TNF-ɑ and IL-6, and marked histological evidence of lung injury (such as alveolar edema, alveolar septal thickening, and neutrophil accumulation) were observed on days 1–2 after acid injury in injured mice, as compared with sham animals. However, these phenomena were not observed in animals treated with sevoflurane (Additional file 1: Fig. S1, Additional file 2: Fig. S1, Additional file 3: Fig. S3).

**Fig. 1:**
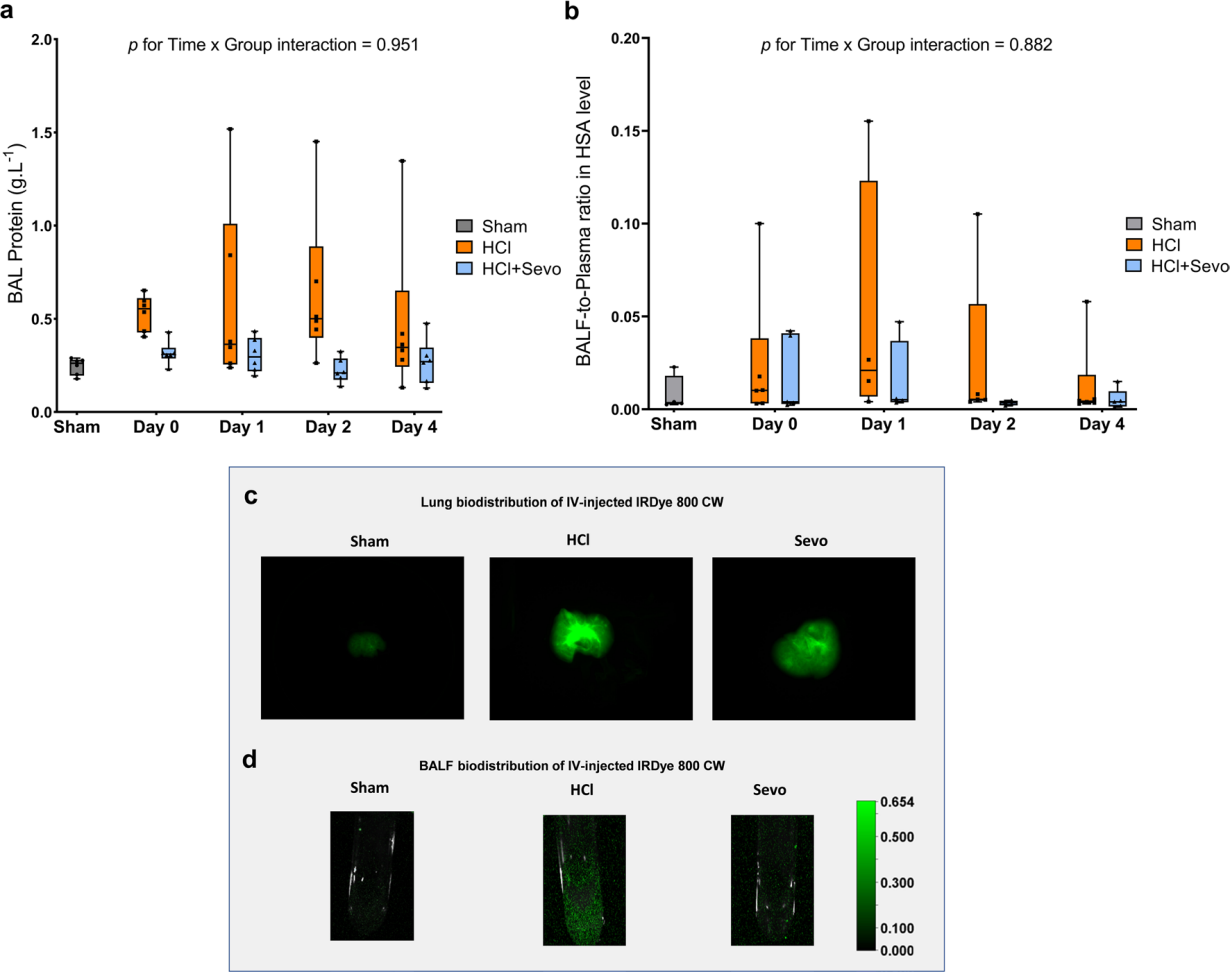
Measures of alveolar-capillary permeability in mice after acid-induced lung injury. **a** Total protein content (in g.L^−1^) of the bronchoalveolar lavage (BAL) fluid and **b** Permeability index, as calculated as the BAL fluid-to-plasma ratio of the human serum albumin (HSA) concentration, in uninjured (Sham), acid-injured (HCl), and acid-injured mice treated with sevoflurane (HCl + Sevo) from day 0 to day 4 after injury. Values are presented as box and whisker plots with medians and interquartile ranges (n = 4–6 per group). Two-way ANOVA tests were performed; no statistical significance was found in **a** and **b**. **c** Representative images of accumulation on day 2 after injury of an intravenously injected, near-infrared fluorescent dye, as reported as relative fluorescence units (RFU), in isolated lungs and **d** in the BAL fluid of uninjured (Sham), acid-injured (HCl), and acid-injured mice treated with sevoflurane (HCl + Sevo)

Alveolar-capillary barrier permeability, as assessed by the permeability index and BAL levels of total proteins, peaked on days 1–2 after injury in acid-injured mice (Fig. 1a and b). This was less marked in animals treated with sevoflurane, although the differences did not reach statistical significance (Time x Group interaction for the permeability index: *p* = 0.88, for BAL total proteins: *p* = 0.95). The extent of edema was further determined through imaging of isolated lung samples and BAL fluid, and fluorescent signals were more intense after acid injury in the control animals than in those treated with sevoflurane (Fig. 1c and d).

### In vivo effects of sevoflurane on mechanisms of lung epithelial integrity

**Fig. 2:**
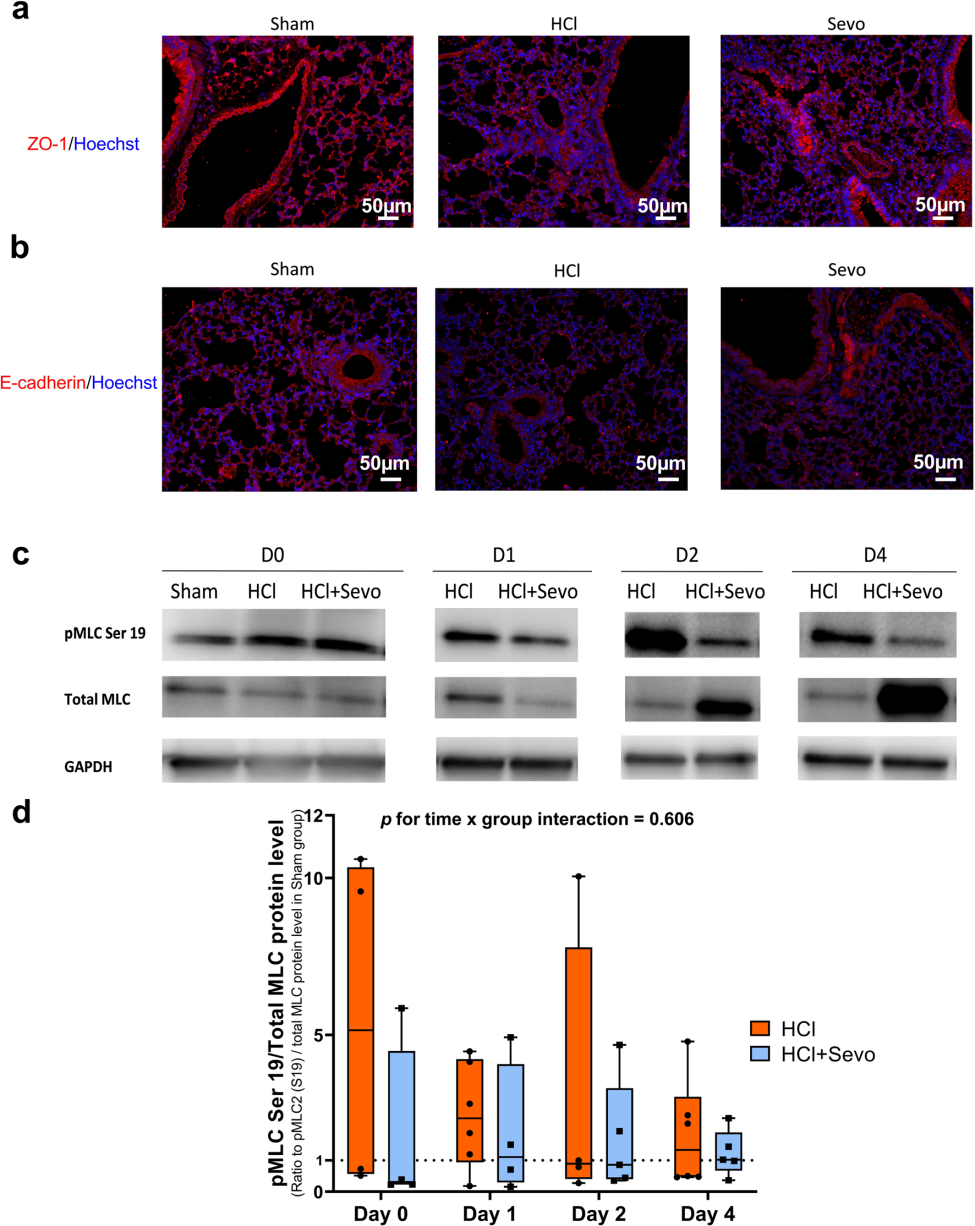
Lung junction proteins zonula occludens (ZO)-1 and E-cadherin and lung myosin light chain 2 (Ser19) phosphorylation (pMLC) in vivo. Immunostaining of lung **a** ZO-1 and **b** E-cadherin in lung tissues from uninjured (Sham), acid-injured (HCl), and acid-injured mice treated with sevoflurane (HCl + Sevo) on day 1 after injury. Tissues were fixed, permeabilized, and stained with ZO-1 and E-cadherin antibodies, followed by A488 secondary antibodies and Hoechst staining. All images were acquired by a fluorescent microscope with a 20× objective. **a** ZO-1 protein is red-stained, and the cell nucleus is blue-stained. **b** E-cadherin protein is red-stained, and the cell nucleus is blue-stained. Scale bar: 50 μm. **c** Western blots of total myosin light chain (MLC) and phosphorylated myosin light chain 2 (Ser19) (pMLC) in lung of uninjured (Sham), acid-injured (HCl), and acid-injured mice treated with sevoflurane (HCl + Sevo) from day 0 to day 4 after injury. **d** Protein expression levels were quantified and standardized by GAPDH protein level, and pMLC (Ser 19) levels were additionally standardized by total MLC level, expressed as ratios to those in Sham animals, and reported as box and whisker plots with medians and interquartile ranges (n = 4–6 per group). Two-way ANOVA test was performed, and no significance was found

Immunostaining studies revealed that ZO-1 and E-cadherin expressions were both markedly decreased in mouse lungs on day 1 after acid injury; in injured animals treated with sevoflurane, however, ZO-1 expression was restored (Fig. 2). This effect of restored ZO-1 expression with sevoflurane following acid injury was confirmed after quantification by Western blot, although there were no differences in ZO-1 mRNA expressions (Additional file 4: Fig. S4, Additional file 5: Fig. S5). There were no between-group differences in E-cadherin expressions assessed by Western blot or RT-qPCR.

Increased pMLC was observed in lungs from mice on day 0 and day 1 after injury, as compared with sham animals. In mice treated with sevoflurane, such an increase was not seen (Fig. 2c, d).

### In vitro effects of sevoflurane on lung epithelial barrier function

**Fig. 3:**
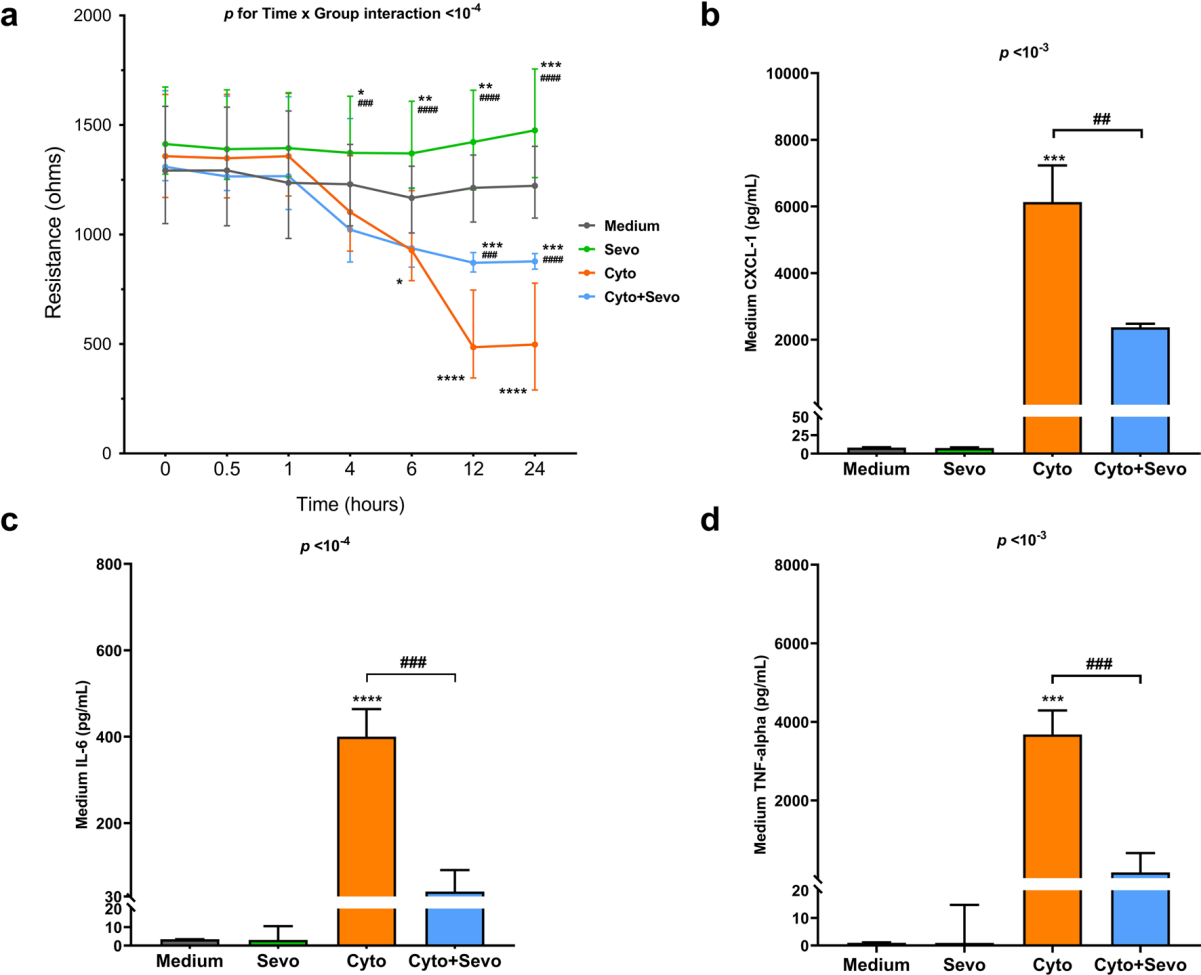
Effects of sevoflurane on electrical resistance and proinflammatory cytokines levels in conditioned medium of mouse lung epithelial (MLE-12) cell monolayer. **a** Electrical resistance of a monolayer of MLE-12 cells was measured at a frequency at 4000 Hz by electric cell-substrate impedance sensing (ECIS) in untreated cells (Medium) or in cells treated for 24 h with cytomix alone (Cyto), sevoflurane alone (Sevo) or with cytomix and sevoflurane (Cyto + Sevo). Results are shown as medians with interquartile ranges (n = 35–40 per group and per timepoint). **b** Medium levels of Chemokine C-X-C motif ligand-1(CXCL-1), **c** Interleukin 6(IL-6) and **d** Tumor necrosis factor-alpha (TNF-α) at 6 h in identical conditions. Results are shown as medians with interquartile ranges (n = 3 per group)**.** Two-way ANOVA test was performed, with post-hoc comparisons if ANOVA results showed significance (compared to the Medium group: *p < 0.05; **p < 0.01; ***p < 10^–3^; ****p < 10^–4^; compared to the Cyto group: ^##^*p* < 0.01; ^###^*p* < 10^–3^; ^####^*p* < 10^–4^)

The electrical resistance of the MLE-12 monolayer, assessed using ECIS, was markedly decreased after treatment with cytomix, which was not found in cells exposed to sevoflurane (Time x Group interaction: *p* < 10^–4^) (Fig. 3a). Post-hoc comparisons revealed significant differences in resistance at 12 and 24 h between cytomix-treated cells exposed or not exposed to sevoflurane (Time x Group interaction: *p* < 10^–3^ and *p* < 10^–4^, respectively).

Treatment with cytomix was associated with increased cytokine release at 6 h, but such a release was significantly prevented by exposure to sevoflurane (Fig. 3b–d).

### In vitro effects of sevoflurane on mechanisms of lung epithelial integrity

Cell viability was significantly decreased in all experimental conditions after treatment with cytomix; exposure to sevoflurane alone had no significant effect on cell viability, compared to the control condition (Additional file 15: Fig. S15).

**Fig. 4:**
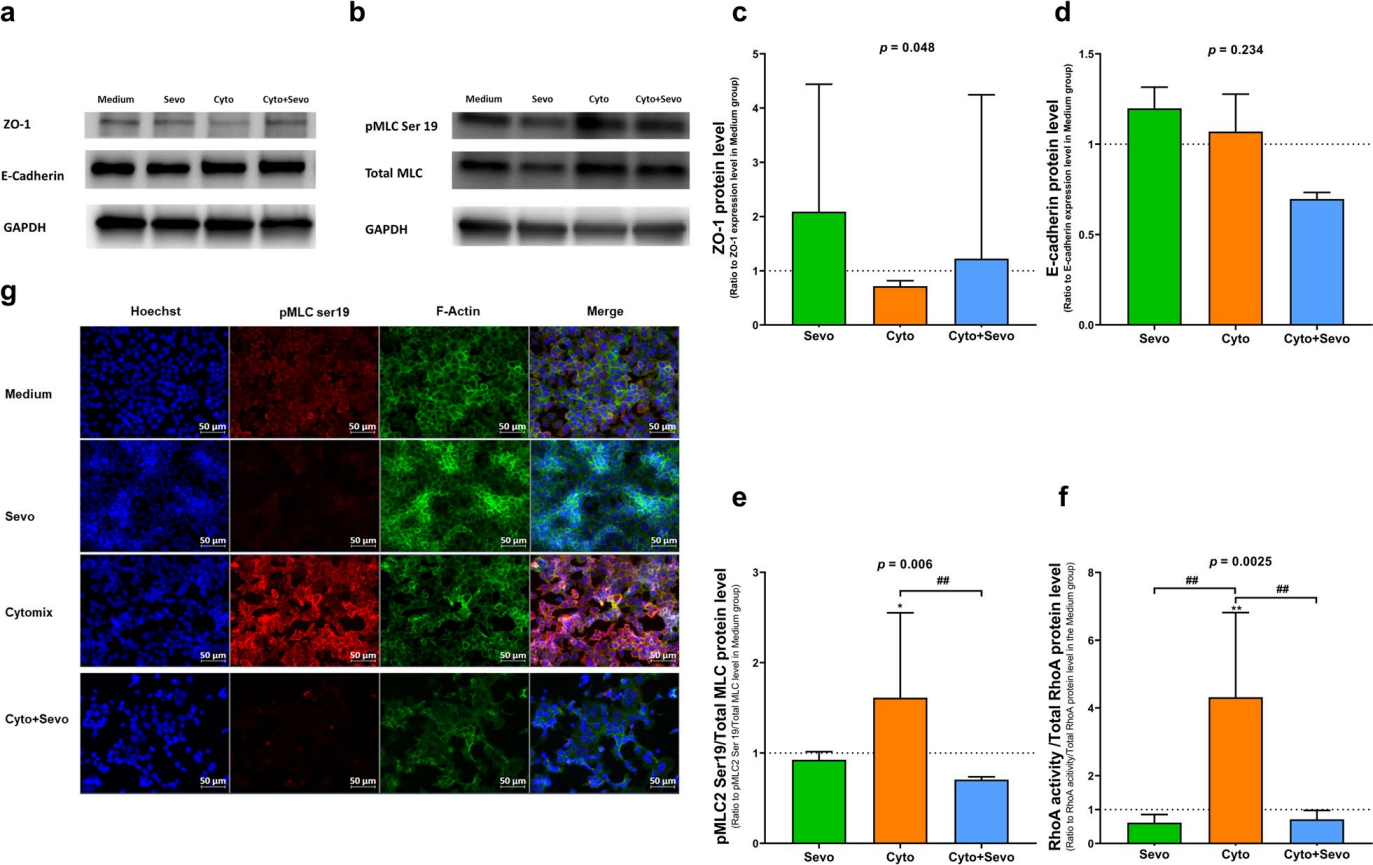
Effects of sevoflurane on junction proteins and RhoA/pMLC/F-actin pathway of mouse lung epithelial (MLE-12) cells. Western blots of** a** ZO-1 and E-cadherin, **b** total myosin light chain (MLC) and phosphorylated myosin light chain 2 (Ser19) (pMLC2) levels at 6 h in untreated MLE-12 cells (Medium) and cells exposed to sevoflurane alone (Sevo), cytomix alone (Cyto) or cytomix and sevoflurane (Cyto + Sevo). **c**–**e** Protein expression levels were quantified and standardized by GAPDH protein level, and pMLC levels were standardized by total MLC levels, expressed as ratios to those in the Medium group. **f** RhoA activity was standardized by total RhoA protein level at 30 min in identical conditions. All results are reported as medians with interquartile ranges. One-way ANOVA was performed, with post-hoc comparisons if ANOVA results showed significance (compared to the Medium group: *p < 0.05; **p < 0.01; compared to the Cyto group: ^##^*p* < 0.01).**g** Immunostaining after 6 h of treatment of pMLC and F-actin was performed in identical conditions. Cells were fixed, permeabilized, and stained with antibodies, followed by A488 secondary antibodies and Hoechst. All images were acquired by fluorescent microscope with a 40× objective. Scale bar: 50 μm

Protein expressions of ZO-1 and E-cadherin decreased at 6 h after treatment with cytomix, but exposure to sevoflurane was associated with a higher expression of ZO-1 protein (Fig. 4a–d and Additional file 6: Fig. S6).

After 6 h of exposure, sevoflurane reduced the cytomix-induced increase in pMLC and actin cytoskeletal rearrangement and contraction, with decreased F-actin staining intensity, in MLE-12 cells (Fig. 4e–g). After 30 min of treatment, RhoA activity was increased in MLE-12 cells treated with cytomix, as compared with those treated with medium only. Exposure of cells to sevoflurane treated with cytomix significantly prevented such an increase (Fig. 4f).

### RAGE-dependent effects of sevoflurane on lung epithelial barrier function in vitro

**Fig. 5:**
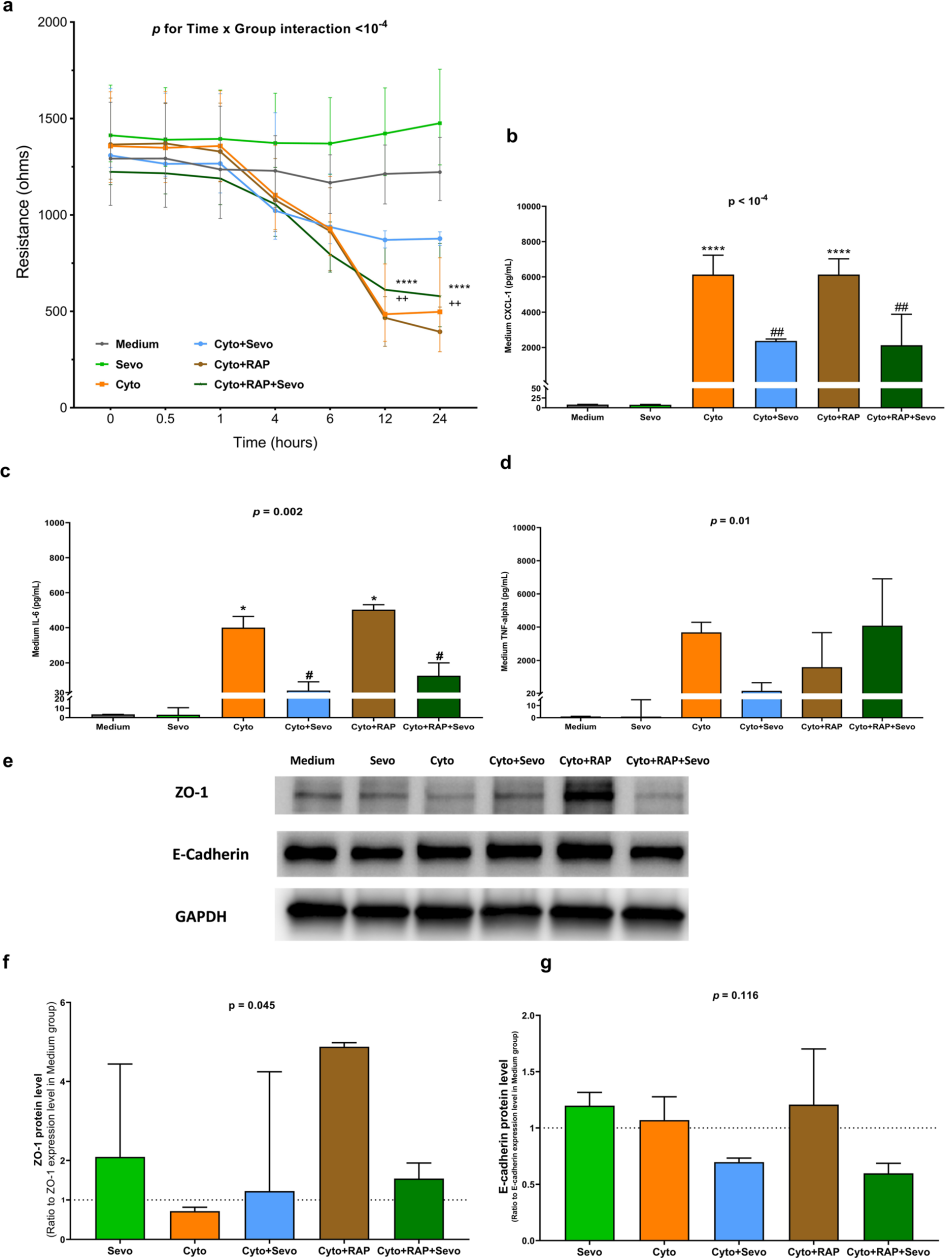
Effects of sevoflurane on lung epithelial barrier function of mouse lung epithelial (MLE-12) cell monolayer, treated or not with RAGE antagonist peptide (RAP). **a** Electrical resistance over 24 h of a monolayer of MLE-12 cells was measured at a frequency at 4000 Hz by electric cell-substrate impedance sensing (ECIS) in untreated cells (Medium) or in cells treated for 24 h with cytomix alone (Cyto), sevoflurane alone (Sevo), cytomix and sevoflurane (Cyto + Sevo), cytomix and RAP (Cyto + RAP) or with cytomix, RAP, and sevoflurane (Cyto + RAP + Sevo). Results are shown as medians with interquartile ranges (n = 35–40 per group and per timepoint). Two-way ANOVA test was performed, with post-hoc comparisons if ANOVA results showed significance (compared to the Medium group: ****p < 10^–4^; compared to the Cyto + Sevo group: ++*p* < 0.01). **b** Medium level of Chemokine C-X-C motif ligand-1(CXCL-1), **c** Interleukin 6(IL-6) and **d** Tumor necrosis factor alpha (TNF-α) at 6 h in identical conditions. **e**) western blots of ZO-1 and E-cadherin at 6 h in identical conditions. **f**) Protein expression levels were quantified and standardized by GAPDH protein level and expressed as ratios to those in the Medium group. Results of **b–f** are shown as medians with interquartile ranges. One-way ANOVA was performed, with post-hoc comparisons if ANOVA results showed significance (compared to the Medium group: *p < 0.05; ****p < 10^–4^; compared to the Cyto group: ^#^*p* < 0.05; ^##^*p* < 0.01)

Treatment with RAP of cells exposed to cytomix did not restore the electrical resistance of MLE-12 cell monolayers in ECIS. Further, when the cells were co-treated with sevoflurane and RAP, the beneficial effect previously found with sevoflurane alone was no longer observed (Fig. 5a). Treatment with RAP alone did not significantly alter the cytomix-induced release of cytokines by MLE-12 cells at 6 h, and RAP alone did not influence the effects of sevoflurane on cytomix-induced cytokine release. However, co-treatment with RAP and sevoflurane was associated with higher medium levels of TNF-ɑ after cytomix, as compared with those after treatment with sevoflurane alone (Fig. 5b–d). Although the experiments with RAP showed no between-group differences in E-cadherin protein levels after 6 h of treatment, RAP was associated with restored protein levels of ZO-1 in cells exposed to cytomix, whether or not they were co-exposed to sevoflurane (Fig. 5e–g).

**Fig. 6:**
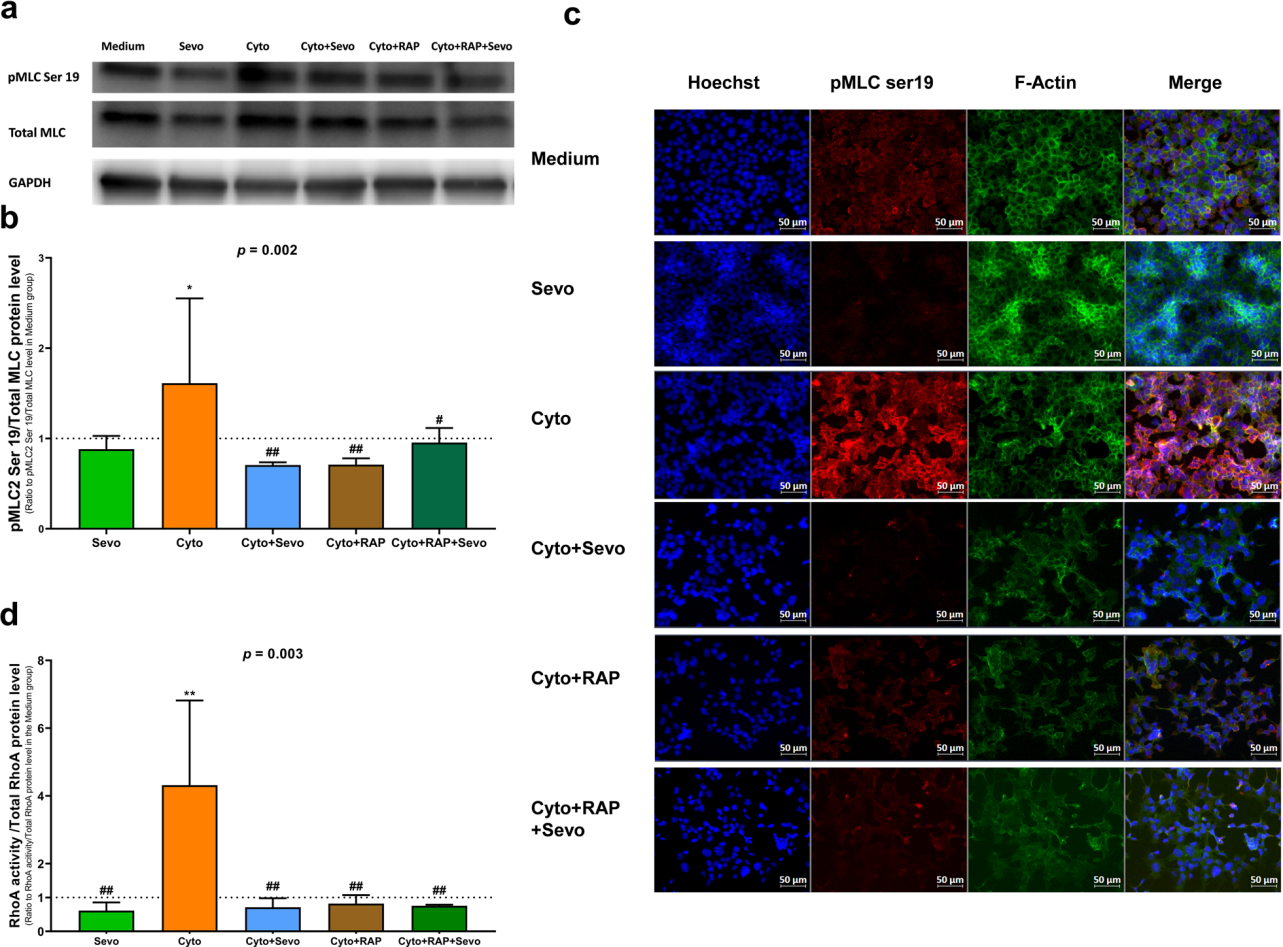
Effects of sevoflurane on RhoA/pMLC/F-actin pathway in mouse lung epithelial (MLE-12) cells, treated or not with RAGE antagonist peptide (RAP). **a** total myosin light chain (MLC) and phosphorylated myosin light chain 2 (Ser19) (pMLC2) levels at 6 h in untreated MLE-12 cells (Medium) and cells exposed to sevoflurane alone (Sevo), cytomix alone (Cyto), cytomix and sevoflurane (Cyto + Sevo), cytomix and RAP (Cyto + RAP) or with cytomix, RAP, and sevoflurane (Cyto + RAP + Sevo). **b** Protein expression levels were quantified and standardized by GAPDH protein level, and pMLC levels were standardized by total MLC levels, expressed as ratios to those in the Medium group, and reported as medians with interquartile ranges. **c** Immunostaining after 6 h of treatment of pMLC and F-actin was performed in identical conditions. Cells were fixed, permeabilized, and stained with antibodies, followed by A488 secondary antibodies and Hoechst. All images were acquired by fluorescent microscope with a 40× objective. Scale bar: 50 μm.** d** RhoA activity was standardized by total RhoA protein level at 30 min in identical conditions. All quantitative results are reported as medians with interquartile ranges. One-way ANOVA was performed, with post-hoc comparisons if ANOVA results showed significance (compared to the Medium group: *p < 0.05; **p < 0.01; compared to the Cyto group: ^#^*p* < 0.05; ^##^*p* < 0.01)

The immunostaining signal and protein quantification based on the Western blot of pMLC were decreased when MLE-12 cells exposed to cytomix were treated with RAP, compared to those who were not treated with RAP (Fig. 6a–c). Although treatment with RAP did not influence the effects of sevoflurane on pMLC levels after exposure to cytomix, F-actin cytoskeletal rearrangement and contraction were increased by RAP in MLE-12 cells exposed to cytomix and sevoflurane (Fig. 6c). Treatment with RAP decreased the cytomix-induced increase in the RhoA activity of MLE-12 cells, as assessed at 30 min. However, RAP did not influence the effects of sevoflurane on RhoA activity after exposure to cytomix (Fig. 6d).

### RAGE-dependent effects of sevoflurane on lung injury and mechanisms of lung epithelial barrier function in vivo

The permeability index and BAL levels of total proteins were lower from day 0 to day 2 after acid injury in RAGE^−/−^ mice than in littermate controls. However, RAGE deletion did not influence the effects of sevoflurane related to permeability indices after injury (Additional file 7: Fig. S7). Similarly, lung accumulation of an intravenous fluorescent tracer on day 2 after injury was decreased in RAGE^−/−^ mice, compared to littermate controls, without additional influence of RAGE deletion on the effects of sevoflurane (Additional file 8: Fig. S8). There were no obvious differences in lung immunostaining of ZO-1 and E-cadherin in RAGE^−/−^ versus wild-type mice on day 1 after injury (Additional file 9: Fig. S9). In RAGE^−/−^ mice, sevoflurane restored both ZO-1 and E-cadherin fluorescent signals after injury in comparison to injured mice not receiving sevoflurane. Overall, ZO-1 and E-cadherin protein levels were overall lower in RAGE^−/−^ animals than in littermate controls (Additional file 10: Fig. S10). In RAGE^−/−^ mice, sevoflurane was not associated with a significant effect on ZO-1 and E-cadherin protein expressions in the lungs over the four-day experiment. Lung pMLC levels peaked on day 1 after acid injury in RAGE^−/−^ mice, a phenomenon abolished by treatment with sevoflurane (Additional file 11: Fig. S11).

RAGE deletion in treated mice was associated with improvements in physiological, inflammatory, and histological features of acid-induced lung injury over time, as compared with wild-type controls (Additional file 12: Fig. S12, Additional file 13: Fig. S13, Additional file 14: Fig. S14). However, the beneficial effect of sevoflurane previously observed in wild-type mice on day 1 after injury in terms of higher PaO_2_/FiO_2_ and decreased BAL levels of IL-6 and TNF-ɑ was not found in RAGE^−/−^ mice.

## Discussion

In this study, we used an in vivo model of acid-induced lung injury in mice and an in vitro model of sterile injury in mouse lung epithelial cells to investigate whether sevoflurane could decrease lung alveolar epithelial permeability through the RhoA/pMLC/F-actin pathway.

In our mouse model of acid-induced ARDS, exposure to sevoflurane was associated with better arterial oxygenation, decreased alveolar inflammation and histological damage, and an attenuated increase in indices lung permeability (such as the permeability index, BAL levels of total protein, and fluorescence assay in isolated lung samples and BAL fluid), in line with previous reports of models of lipopolysaccharide-induced lung injury in rats and a pig model of surfactant saline lavage [13, 15, 16, 38]. Alterations of inter-epithelial tight and adherens junction proteins are major contributors to lung epithelial barrier dysfunction in ARDS [3, 39]. Our study focused on the integrity of the epithelial barrier function, finding decreased indices of permeability and preserved epithelial structures in cells and mice exposed to sevoflurane after injury. Sevoflurane increased the protein expression of ZO-1 in our in vivo and in vitro models, further supporting previous findings of alleviated lung permeability due to the upregulation of occludin and ZO-1 with sevoflurane preconditioning before ischemia–reperfusion in rats [40]. In another double-hit mouse model, isoflurane restored epithelial tight junction integrity and increased ZO-1 levels [21]. However, no effect of sevoflurane on E-cadherin was observed in our study, contrasting with previous findings on human lung microvascular endothelial cells injured by LPS and on colon carcinoma cell lines [22, 41]. In particular, our study provides novel evidence supporting the molecular mechanisms of the effects of sevoflurane on lung epithelial barrier function after injury. Notably, sevoflurane was associated with decreased lung levels of pMLC and decreased actin cytoskeletal rearrangement after injury in vivo and in vitro. Sevoflurane also decreased cytomix-induced RhoA activity in vitro, suggesting that sevoflurane could decrease lung epithelial permeability through inhibition of the RhoA/pMLC/F-actin cytoskeleton pathway, as also suggested by studies on other cell types [22, 42–46].

In vivo, acid-injured RAGE^−/−^ mice unexposed to sevoflurane had better oxygenation levels, decreased lung permeability, and improved inflammatory response compared to littermate wild-type animals, as previously reported for RAGE inhibition strategies using recombinant sRAGE as a decoy receptor or an anti-RAGE monoclonal antibody as an antagonist [31]. RAGE^−/−^ mice received the same benefits from sevoflurane as littermate controls in terms of indices of lung alveolar-capillary permeability. However, in contrast to wild-type mice, the RAGE^−/−^ animals did not exhibit the effects of improved arterial oxygenation and decreased BAL levels of IL-6 and TNF-ɑ observed with sevoflurane, suggesting that RAGE could play a mediating role in these specific effects of sevoflurane but less influence on sevoflurane-induced changes in permeability. In vitro, treatment with RAP decreased cytomix-induced RhoA activity in MLE-12 cells and alleviated the beneficial effects of sevoflurane on electrical resistance and actin cytoskeletal rearrangement of MLE-12 cells exposed to cytomix.

Our study has limitations. First, we used in vitro and vivo models of injury from sterile causes, and thus our findings may not be generalizable to other settings. Second, we used MLE-12 cells (i.e., type-2-like, tumor-derived epithelial cells), and future validation studies on primary mouse or human alveolar epithelial cells are warranted. Third, our mechanistic analyses focused on a short time frame since we hypothesized this would be more relevant for studying the RhoA/pMLC/F-actin signaling pathway, which was only feasible in vitro. Fourth, although we investigated lung epithelial permeability using modern and relevant approaches focusing on the barrier function and intercellular junctions, the extent of lung epithelial injury also depends on other important mechanisms, such as cell death [41], wound healing or fluid and ion clearance. Further investigation is needed to determine whether sevoflurane affects such mechanisms.

Our study also has several strengths. We used a mouse model of direct lung epithelial injury over multiple days [27, 30, 31] and an in vitro model of alveolar epithelial injury [34, 47], which are validated and have translational value. In addition, the sevoflurane concentrations used in our study are similar to those used in clinical practice for deep sedation, which is often required in the early management of clinical ARDS [12, 48], and in a large multicenter clinical trial [20]. Further, a better description of the mechanisms of lung epithelial injury, among other features of ARDS pathogeny, is important to identify endotypes within ARDS (i.e., subgroups with distinct biological or functional features) and to inform the future development of more targeted, endotype-based therapies for ARDS [49–51].

In conclusion, sevoflurane was shown to have protective effects on lung epithelial permeability and epithelial junction proteins in experimental models of sterile ARDS. These protective effects could be explained, at least in part, by the inhibition of increased RhoA activity and pMLC as well as actin cytoskeleton rearrangement following lung epithelial injury. Further studies are needed to determine whether the RAGE pathway mediates some of these effects.

## Supplementary Information

**Additional file 1: Figure S1.** Arterial oxygen tension (PaO_2_)/inspiratory oxygen fraction (FiO_2_) in mice after acid-induced lung injury. Arterial oxygen tension (PaO2)/inspiratory oxygen fraction (FiO2) of uninjured (Sham), acid-injured (HCl), and acid-injured mice treated with sevoflurane (HCl + Sevo) from day 0 to day 4 after injury. Values are presented as box and whisker plots with medians and interquartile ranges. Two-way ANOVA tests were performed, and post-hoc comparisons were performed if ANOVA results showed significance (compared to the Sham group: *p < 0.05; ****p < 10^–4^; compared to the HCl group: ^###^*p* < 10^–3^).

**Additional file 2: Figure S2.** Bronchoalveolar lavage fluid (BALF) proinflammatory cytokines levels in mice after acid-induced lung injury. BALF level of** a**) Chemokine C-X-C motif ligand-1(CXCL-1), **b**) Interleukin 6(IL-6) and **c**) Tumor necrosis factor alpha (TNF-α) of uninjured (Sham), acid-injured (HCl), and acid-injured mice treated with sevoflurane (HCl + Sevo) from day 0 to day 4 after injury. Values are presented as box and whisker plots with medians and interquartile ranges. Two-way ANOVA tests were performed, and post-hoc comparisons were performed if ANOVA results showed significance (compared to the WT_Sham group: ****p < 10^–4^; compared to the WT_HCl group: ^##^*p* < 0.01).

**Additional file 3: Figure S3.** Histological features of lung injury in mice after acid-induced lung injury. **a**) Lung histological stainings and **b**) Lung injury scores of uninjured (Sham), acid-injured (HCl), and acid-injured mice treated with sevoflurane (HCl + Sevo) from day 0 to day 4 after injury. Values are presented as box and whisker plots with medians and interquartile ranges. Two-way ANOVA tests were performed, and post-hoc comparisons were performed if ANOVA results showed significance. Two-way ANOVA tests were performed, and post-hoc comparisons were performed if ANOVA results showed significance (compared to the Sham group: ****p < 10^–4^; compared to the HCl group: ^###^*p* < 10^–3^; ^####^*p* < 10^–4^).

**Additional file 4: Figure S4.** Western blots of lung junction proteins zonula occludens (ZO)-1 and E-cadherin in vivo. **a**) Western blots of ZO-1 and E-cadherin in lung tissues from uninjured (Sham), acid-injured (HCl), and acid-injured mice treated with sevoflurane (HCl + Sevo) from day 0 to day 4 after injury. **b**) ZO-1 and **c**) E-cadherin expression levels were quantified and standardized by GAPDH protein level, expressed as ratios to those in sham animals, and represented as box and whisker plots with medians and interquartile ranges. Two-way ANOVA tests were performed, with post-hoc comparisons if ANOVA results showed significance (compared to the Sham group: ****p < 10^–4^; compared to the HCl group: ^###^*p* < 10^–3^).

**Additional file 5: Figure S5.** Lung mRNA levels of lung junction protein zonula occludens (ZO)-1 and E-cadherin in vivo. **a)** ZO-1 and **b)** E-cadherin mRNA levels measured by RT-qPCR in lung tissues from uninjured (Sham), acid-injured (HCl), and acid-injured mice treated with sevoflurane (HCl + Sevo) from day 0 to day 4 after injury. mRNA levels were calculated by the delta-delta Ct method standardized with the housekeeping gene GAPDH. mRNA levels are expressed as ratios to those in sham animals, represented as box and whisker plots with medians and interquartile ranges. Two-way ANOVA test was performed, and no significance was observed.

**Additional file 6: Figure S6.** mRNA levels of lung junction protein zonula occludens (ZO)-1 and E-cadherin in mouse lung epithelial (MLE-12) cells. **a)** ZO-1 and **b)** E-cadherin mRNA levels measured by RT-qPCRin untreated MLE-12 cells (Medium) and cells exposed to sevoflurane alone (Sevo), cytomix alone (Cyto) or cytomix and sevoflurane (Cyto + Sevo). mRNA levels were calculated by the delta-delta Ct method standardized with the housekeeping gene GAPDH. mRNA levels are expressed as ratios to those in the Medium group, represented as medians and interquartile ranges. Two-way ANOVA test was performed, with post-hoc comparisons if ANOVA results showed significance. (compared to the Medium group: **p < 0.01; compared to the Cyto group: ^#^p < 0.05).

**Additional file 7: Figure S7.** Measures of alveolar-capillary permeability in RAGE-/- and littermate wild-type mice after acid-induced lung injury. **a**) Total protein content (in g.L-1) of the bronchoalveolar lavage (BAL) fluid and **b**) Permeability index, as calculated as the BAL fluid-to-plasma ratio of the human serum albumin (HSA) concentration, in wild-type (WT) or RAGE-/- uninjured (Sham), acid-injured (HCl), and acid-injured mice treated with sevoflurane (HCl + Sevo) from day 0 to day 4 after injury. Values are presented as box and whisker plots with medians and interquartile ranges. Two-way ANOVA tests were performed, and no significance was observed.

**Additional file 8: Figure S8.** Effects of sevoflurane on lung accumulation of an intravenous fluorescent tracer in RAGE^−/−^ and wild-type mice on day 2 after acid-induced injury. Representative images of accumulation on day 2 after injury of an intravenously-injected, near-infrared fluorescent dye, as reported as relative fluorescence units (RFU), a) in isolated lungs and **b**) in the bronchoalveolar lavage fluid from RAGE^−/−^ (KO) and littermate wild-type (WT) mice: uninjured (Sham), acid-injured (HCl), and acid-injured treated with sevoflurane (HCl + Sevo).

**Additional file 9: Figure S9.** Immunostaining of lung junction proteins Zonula Occludens (ZO)-1 and E-cadherin in RAGE^−/−^ and wild-type mice on day 1 after acid-induced injury. Immunostaining of lung **a**) ZO-1 and **b**) E-cadherin in lung tissues from RAGE^−/−^ (KO) and littermate wild-type (WT) mice, either uninjured (Sham), acid-injured (HCl) or acid-injured treated with sevoflurane (HCl + Sevo), on day 1 after injury. Tissues were fixed, permeabilized, and stained with ZO-1 and E-cadherin antibodies, followed by A488 secondary antibodies and Hoechst staining. All images were acquired by a fluorescent microscope with a 20 × objective. **a**) ZO-1 protein is red-stained, and the cell nucleus is blue-stained. **b**) E-cadherin protein is red-stained, and the cell nucleus is blue-stained. Scale bar: 50 μm.

**Additional file 10: Figure S10.** Western blots of lung junction proteins zonula occludens (ZO)-1 and E-cadherin in lung tissues from RAGE^−/−^ and wild-type mice after acid-induced injury.** a**) Western blots of ZO-1 and E-cadherin in lung tissues from RAGE^−/−^ (KO) and littermate wild-type (WT) mice, either uninjured (Sham), acid-injured (HCl) or acid-injured treated with sevoflurane (HCl + Sevo), from day 0 to day 4 after injury. **b**) ZO-1 and **c**) E-cadherin expression levels were quantified and standardized by GAPDH protein level, expressed as ratios to those in sham WT animals, and represented as box and whisker plots with medians and interquartile ranges. Two-way ANOVA tests were performed, with post-hoc comparisons if ANOVA results showed significance (compared to the WT_Sham group: ****p < 10^–4^; compared to the WT_HCl group: ^##^p < 0.01).

**Additional file 11: Figure S11. **Myosin Light Chain phosphorylation (Ser 19) in lung tissues from RAGE^−/−^ and wild-type mice after acid-induced injury.** a**) Western blots of total myosin light chain (MLC) and phosphorylated myosin light chain 2 (Ser19) (pMLC) in lung tissues from RAGE^−/−^ (KO) and littermate wild-type (WT) mice, either uninjured (Sham), acid-injured (HCl) or acid-injured treated with sevoflurane (HCl + Sevo), from day 0 to day 4 after injury. **b**) Protein expression levels were quantified and standardized by GAPDH protein level, and pMLC levels were standardized by total MLC levels and expressed as ratios to those in the Medium group. Two-way ANOVA test was performed, and no significance was observed.

**Additional file 12: Figure S12.** Arterial oxygen tension (PaO_2_)/inspiratory oxygen fraction (FiO_2_) in mice after acid-induced lung injury. Arterial oxygen tension (PaO_2_)/inspiratory oxygen fraction (FiO_2_) of littermate control (WT) and RAGE^−/−^ mice, uninjured (Sham), after acid-induced injury (HCl) or after acid-induced injury with treatment by sevoflurane (HCl + Sevo) from day 0 to day 4 after injury. Values are presented as box and whisker plots with medians and interquartile ranges. Two-way ANOVA tests were performed, and post-hoc comparisons were performed if ANOVA results showed significance (compared to the WT_Sham group: *p < 0.05; ***p < 10^–3^; ****p < 10^–4^; compared to the WT_HCl group: ^###^*p* < 10^–3^; compared to the WT_HCl + Sevo group: + , *p* < 0.05).

**Additional file 13: Figure S13.** Bronchoalveolar lavage fluid (BALF) proinflammatory cytokines levels in mice after acid-induced lung injury. BALF level of** a**) Chemokine C-X-C motif ligand-1(CXCL-1), **b**) Interleukin 6(IL-6) and **c**) Tumor necrosis factor alpha (TNF-α) of littermate control (WT) and RAGE^-/-^ mice, uninjured (Sham), after acid-induced injury (HCl) or after acid-induced injury with treatment by sevoflurane (HCl + Sevo) from day 0 to day 4 after injury. Values are presented as box and whisker plots with medians and interquartile ranges. Two-way ANOVA tests were performed, and post-hoc comparisons were performed if ANOVA results showed significance (compared to the WT_Sham group: ****p < 10^–4^; compared to the WT_HCl group: ^##^*p* < 0.01; compared to the WT_HCl + Sevo group: + , *p* < 0.05).

**Additional file 14: Figure S14.** Histological features of lung injury in mice after acid-induced lung injury. **a**) Section images and **b**) Lung injury scores of littermate control (WT) and RAGE-/- mice, uninjured (Sham), after acid-induced injury (HCl) or after acid-induced injury with treatment by sevoflurane (HCl + Sevo) from day 0 to day 4 after injury. Values are presented as box and whisker plots with medians and interquartile ranges. Two-way ANOVA tests were performed, and post-hoc comparisons were performed if ANOVA results showed significance (compared to the WT_Sham group: *p < 0.05; ****p < 10^–4^; compared to the WT_HCl group: ^###^*p* < 10^–3^; ^####^*p* < 10^–4^; compared to the KO_HCl group: ++++ , *p* < 10^–4^).

**Additional file 15: Figure S15.** Cell viability of experimental conditions. Cell viability at 6 h in untreated MLE-12 cells (Medium) and cells exposed to sevoflurane alone (Sevo), cytomix alone (Cyto), cytomix and sevoflurane (Cyto + Sevo), cytomix and RAP (Cyto + RAP) or with cytomix, RAP, and sevoflurane (Cyto + RAP + Sevo). Cell viability of all conditions is referred to the medium group as 100%. Results are shown as mean with SD (n = 4 per group). One-way ANOVA was performed, with post hoc comparisons, if ANOVA results showed significance (compared to the Medium group: ****p < 10^–4^).

## Data Availability

The research protocols and analysis plans are available in the current manuscript. All study data will be available at time of publication to researchers who provide a methodologically sound and ethically approved proposal, for any purpose of analysis. A data use agreement will be required before the release of participant data and institutional review board approval as appropriate.

## Abbreviations

ARDS: Acute respiratory distress syndrome
BAL: Bronchoalveolar lavage
ECIS: Electric cell-substrate impedance sensing
IL-6: Interleukin-6
LPS: Lipopolysaccharide
MLC: Myosin light chain
MLE-12: Mouse lung epithelial-12
pMLC: Phosphorylated myosin light chain
RAGE: Receptor for advanced glycation end products
RAP: RAGE antagonist peptide
RhoA: Ras homolog family member A
TNF-α: Tumor necrosis factor alpha
ZO-1: Zonula occludens-1
